# Animal Rabies in Kazakhstan: Stable Endemicity, Surveillance Pitfalls, and Priority Actions

**DOI:** 10.3390/pathogens14111079

**Published:** 2025-10-23

**Authors:** Sarsenbay K. Abdrakhmanov, Asem Zh. Abenova, Aizada A. Mukhanbetkaliyeva, Fedor I. Korennoy, Andres M. Perez

**Affiliations:** 1 Institute of Animal Science and Veterinary, S. Seifullin Kazakh Agro Technical Research University, Astana 010011, Kazakhstan; asem.abenova.1993@mail.ru (A.Z.A.); aizada.1970@mail.ru (A.A.M.); 2 Federal Centre for Animal Health (FGBI ARRIAH), Vladimir 600901, Russia; korennoy@arriah.ru; 3 Federal Research Center for Virology and Microbiology, Branch in Nizhny Novgorod, Nizhny Novgorod 603950, Russia; 4 Center for Animal Health and Food Safety, College of Veterinary Medicine, University of Minnesota, St. Paul Campus, St. Paul, MN 55108, USA; aperez@umn.edu

**Keywords:** rabies, Kazakhstan, One Health, vaccination, control, epidemiology

## Abstract

Rabies is endemic in Kazakhstan, with the primary reservoirs being wild canids, such as foxes and dogs, maintaining distinct sylvatic and urban cycles. This paper outlines three high-return priorities for rabies control in the country, informed by the epidemiological patterns of the disease, the national regulatory framework (Order No. 7-1/587), and evidence on the knowledge, attitudes, and practices (KAP) of the Kazakh population. The three priorities are (a) transition into a One Health, real-time surveillance system featuring standardized digital reporting and GIS-guided interventions; (b) implementation of biannual oral rabies vaccination (ORV) of foxes in high-risk districts, incorporating mandatory quality assurance (via tetracycline biomarkers and/or serology) aligned with EU/EFSA standards; and (c) adopt an urban strategy focused on dogs to increase vaccination coverage and reduce delays in human post-exposure prophylaxis (PEP). These measures align with the WOAH Terrestrial Code and the “Zero by 30” roadmap, leveraging existing national assets like risk maps and laboratory capacity, such as dFAT, RT-PCR, and sequencing. Kazakhstan’s predictable rabies pattern allows for targeting district-level strategies and transparent measurement of risk reduction, contingent on enforcing standardized reporting and rigorous quality assurance programs. The opinions introduced in this paper are based on the scientific evidence collected in Kazakhstan over the last decade and summarize the need for urgent actions to promote rabies control in the country.

## 1. Introduction

Rabies is a disease of mammals considered to be almost uniformly fatal once clinical signs appear, yet it is fully preventable with timely post-exposure prophylaxis (PEP) [1,2]. The global “Zero by 30” initiative targets the elimination of dog-mediated human rabies deaths by 2030, providing a unifying programmatic objective and a shared monitoring language across sectors [3]. Within the veterinary domain, the WOAH Terrestrial Animal Health Code (Rabies) defines surveillance, vaccination, and movement-control standards that national programs can operationalize and audit against [4].

Kazakhstan experiences persistent, endemic transmission with recurrent geographic and seasonal clustering rather than episodic nationwide waves. Rabies cases have been documented since 1914 in the country, with the first case reported in the Turgay region [5]. Over the last decade (2013–2025), surveillance data recorded 985 animal cases, with 25 human deaths reported in 2015–2024, a pattern suggesting entrenched circulation despite ongoing control measures [6,7,8,9]. The economic burden remains substantial. Losses in livestock, PEP expenditure, and surveillance/response costs cumulatively reach significant annual totals for endemic countries in Asia; benchmark estimates (contextualizing Kazakhstan’s setting) highlight the high cost-effectiveness of coordinated vaccination and timely PEP [10,11].

From a biological standpoint, essential for program design, the rabies virus (RABV) of the genus *Lyssavirus* (family *Rhabdoviridae*) is transmitted predominantly via saliva through bites; the incubation period typically ranges 1–3 months with centripetal neuroinvasion and limited early immune visibility. In humans, once hydrophobia/aerophobia, agitation, or paralysis manifest, death usually ensues within days to weeks; in animals, field presentation is classically furious or paralytic [2,12].

Kazakhstan’s regulatory backbone—Order No. 7-1/587—aligns implementation (e.g., vaccination, diagnostics, outbreak response) and interfaces with international guidance [13,14]. Laboratory capacity (dFAT, RT-PCR, sequencing) and national risk mapping have expanded in recent years, creating the preconditions for district-level targeting and standardized quality assurance (QA) loops [7,15,16,17]. Complementary behavioral evidence from KAP studies—both regional analogs and emerging Kazakhstan-specific data—underscores gaps in awareness, vaccination uptake, and health-seeking behavior that directly affect the timeliness of PEP and vaccination coverage in dogs [11,18].

## 2. Distribution of Animal Rabies in Kazakhstan

Rabies in Kazakhstan represents an endemic transmission pattern characterized by a stable baseline incidence and recurring, analyzable clusters, rather than episodic, nationwide outbreaks. The 985 animal cases reported between 2013 and 2025 followed a consistent spatiotemporal pattern that supports targeted, district-level intervention strategies.

Risk stratification using the Forest-Based Time Series Forecast modeling approach [7] identified three distinct categories of districts:

- High-risk districts (n = 7), primarily located in border regions (e.g., West Kazakhstan, East Kazakhstan, Pavlodar, Jetisu, Zhambyl oblasts);
- Medium-risk (vaccination-focused) districts (n = 118);
- Low-risk (enhanced monitoring) districts (n = 51).

**Figure 1:**
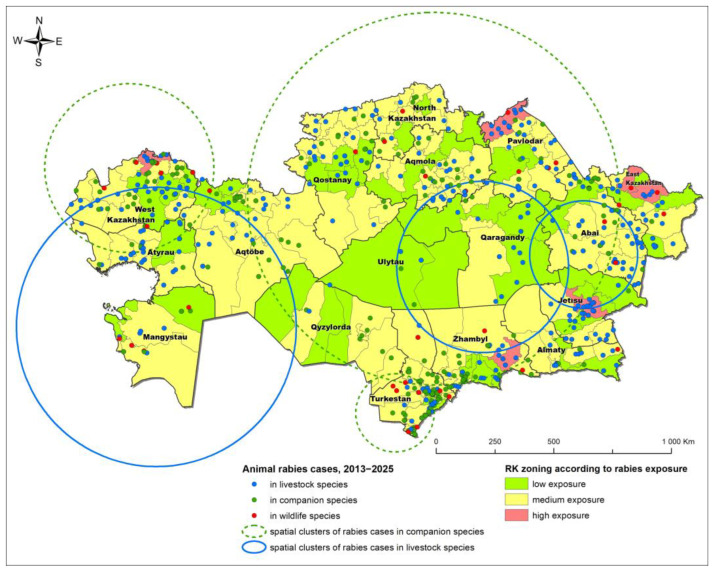
Animal rabies distribution and risk zoning in Kazakhstan, 2013–2025.

Spatiotemporal analysis using a space-time cube and spatial scan statistics revealed distinct patterns of rabies clustering. Urban areas with high dog populations, such as Turkestan and Shymkent, showed strong clustering with observed-to-expected (O/E) ratios exceeding 2.0 (Figure 1). In contrast, rural spillover clusters in regions like East Kazakhstan and Pavlodar were consistent with fox-to-livestock transmission pathways [6,7].

Seasonality in rabies cases is particularly evident in cattle, with peaks coinciding with spring and summer grazing periods. In contrast, rabies in dogs occurs throughout the year, indicating a more continuous transmission cycle.

By species (2013–2025, n = 985), cattle accounted for most (55.8%; n = 550) cases, followed by dogs and cats at 38.4% (n = 378), and wild canids (foxes and wolves) at 5.8% (n = 57). Data suggest a higher incidence of rabies in livestock in the eastern regions of the country compared to the south, where cases in companion animals are relatively more prevalent [6,7]. Molecular characterization of isolates revealed that all Kazakhstani strains fall within the Cosmopolitan clade, suggesting the presence of entrenched local transmission cycles rather than repeated introductions of new viral strains [15].

Reports of lyssaviruses in bat species such as *Eptesicus serotinus* and *Vespertilio murinus* justify the implementation of targeted chiropteran surveillance, although the relatively low number of such cases suggests that bats may not serve as the primary reservoir for the terrestrial rabies cycles.

The long-term stability of rabies spread in Kazakhstan highlights the importance of integrating environmental, veterinary, and social determinants into comprehensive control strategies. The recent 68.1% decline in livestock rabies cases during the first half of 2025 offers a valuable opportunity to assess the effectiveness of ongoing control programs. However, the persistent clustering of livestock cases in the Qostanay region signals underlying structural vulnerabilities that require tailored, localized responses. In short, the stability of rabies transmission is not merely a challenge—it also presents an opportunity for more focused and effective management through advanced analytical tools and intersectoral collaboration [6].

## 3. Control and Monitoring Efforts

Rabies control in Kazakhstan operates within a system that evolved from Soviet-era foundations and adapted to modern demands. The core surveillance strategy remains predominantly passive: veterinary services respond to reports of suspect animal cases or human exposures, cull suspect animals, and confirm diagnoses via dFAT or RT-PCR testing at reference laboratories. While this reactive approach complies with WOAH Terrestrial Manual standards, it falls short in the detection of incubation infections—particularly in remote areas, resulting in a systematic underestimation of prevalence [6].

Since the 2000s, elements of active surveillance, such as targeted sampling in peri-focal areas, have been introduced, though the passive framework continues to dominate the surveillance scheme [17]. A significant modernization occurred in 2024–2025 with the integration of GIS-assisted forecasting, which has reduced the median predicted incidence to below one case per district; however, the accuracy of these forecasts remains contingent on the completeness of underlying data [6,7].

These surveillance activities are governed by Order No. 7-1/587 (29 June 2015), which mandates the annual parenteral vaccination of dogs, cats, and livestock in declared endemic areas. However, the parameters used for zone recognition are not clearly defined, making it difficult to distinguish between areas of genuinely low rabies incidence and those with inefficient surveillance, which is a significant limitation in Kazakhstan and other rabies-infected countries.

Some important features of the administration of the vaccination program in Kazakhstan are summarized in Table 1. Between 2013 and 2015, Kazakhstan’s parenteral vaccination program administered approximately 4.7 million doses annually at a cost of USD 1.7 million, with cattle vaccination (USD 0.35/dose) accounting for USD 1.4 million of this total. Distribution was prioritized, with 60% of doses allocated to the high-risk south and east regions and 20% each to the north and west. The program targets ≥70% coverage to achieve herd immunity, consistent with WOAH/WHO guidance [3]. Alongside domestic animal vaccination, an Oral Rabies Vaccination (ORV) program targeting foxes has been operational since the early 2000s. Initially deploying 736,000 baits annually (at USD 0.97/bait, totaling USD 719,000), the program has struggled with bait density. The recommended rate of 25–30 baits/km^2^ was seldom achieved, and campaigns limited to November are suboptimal given disease seasonality, as noted in the WOAH Terrestrial Code [4].

From 2016 to 2023, parenteral vaccination remained stable at 4.5–5.0 million doses/year, with up to 65% directed to endemic southern and eastern border oblasts. This sustained effort paralleled a decline in animal cases from 120 (2016) to 70 (2023) [10]. The ORV program expanded significantly, growing from 736,000 baits in 2015 to 1.2 million annually between 2020 and 2023, and incorporated the more effective VRC-RZ2 vaccine [15].

Despite these efforts, challenges persist. Rural dog vaccination coverage remained low at 20–25%, sustaining 6–8 endemic foci annually in regions like Qostanay and East Kazakhstan [9]. This, combined with the need to provide PEP for 60,000–70,000 people annually, underscores the ongoing transmission risk. While these combined measures successfully reduced human deaths to fewer than 3 per year by 2023, they have not yet fully interrupted the transmission cycle [17].

Recent data from 2024 to 2025 highlights this uneven progress: Qostanay’s dog coverage was only 21.1%, with six active outbreaks, whereas a national intensification of measures correlated with a 68.1% decline in livestock morbidity in 2024 as compared to 73 cases in 2013 [17]. Program innovations, such as the VRC-RZ2 vaccine (reporting 180-day immunity) [18] and recombinant N-protein-based diagnostics [19], offer promising tools for a more effective future strategy.

## 4. Epizootic Situation of Rabies in Countries Neighboring Kazakhstan

An analysis of the rabies situation in countries neighboring Kazakhstan demonstrates the importance of transboundary transmission of the disease and the need for strengthening regional collaborations.

No outbreaks have been registered in China, the Russian Federation, Uzbekistan, or Kyrgyzstan at the World Organization for Animal Health World Animal Health Information System (WOAH WAHIS) since 2009 [20]. However, national data from those countries describe the presence of the disease. Specifically, national surveillance data indicate continuous virus circulation in China. From 2004 to 2024, 331 animal cases were laboratory-confirmed, primarily among dogs (47%) and cattle (43%) [21]. The main burden of the disease falls on the northern provinces. Similarly, according to the Russian Federal Service for Veterinary and Phytosanitary Surveillance (Rosselkhoznadzor), at least 200 cases were detected in 15 regions from January to October 2025 alone [22,23]. The main reservoir is wild animals, primarily foxes [24]. Annually, 20–30 human rabies cases are registered in Uzbekistan, with stray dog bites being the primary source of infection [25]. An outbreak was recorded in the Jizzakh Region in 2025 [26]. Stray dogs constitute 80–90% of the virus reservoir [27]. Finally, there is a substantial number of animal bite incidents in Kyrgyzstan, where from January to August 2025, a total of 2318 such requests were registered in Bishkek, a 20% increase compared to the same period in 2024 [28]. The main virus reservoir is dogs [29].

These findings, along with the limited data available at international organizations’ websites, demonstrate the need for international coordination to support disease control in the region.

## 5. Knowledge and Attitudes of the Population and Factors Contributing to the Spread

Knowledge, attitudes, and practices (KAP) are critical determinants of rabies control in endemic settings like Kazakhstan, where up to 99% of human infections originate from dog bites [5]. A 2025 cross-sectional survey in Aktobe and Oral (Uralsk) among cattle producers quantified significant KAP gaps: while 52% of respondents correctly identified post-exposure prophylaxis (PEP) requirements, only 23% reported routinely vaccinating their animals. The primary barriers were financial constraints, distance to veterinary services, and low perceived risk [11]. Notably, 87% held favorable attitudes towards prevention, indicating a latent readiness for action if structural obstacles were addressed.

Non-compliance with mandatory vaccination under Order No. 7-1/587 remains a core driver of sustained transmission [13]. Annual vaccination is frequently skipped due to out-of-pocket costs and limited clinic access, perpetuating viral circulation in the south and east. Delays in seeking medical care further elevate risk; in some rural communities, traditional remedies are attempted before PEP—a dangerous practice for a disease that is almost 100% fatal without timely intervention [5]. This awareness-to-action gap was starkly illustrated in 2025 by vaccination refusals and a rabies-associated fatality in East Kazakhstan and Abai oblasts [9].

Exposure to wildlife adds another layer of complexity. Producers in eastern and western rangelands report encounters with potentially rabid foxes and wolves, sometimes involving direct contact due to underestimated risk, which facilitates spillover to livestock and secondary human exposure. Stray dogs remain the principal reservoir, disproportionately threatening children—a demographic that accounts for roughly 40% of global rabies deaths, a pattern likely mirrored in Kazakhstan despite under-reporting [5]. Vast territories, high livestock density, and sparse veterinary access compound these risks. Comparative analyses suggest that only 18.9% of the surveyed population demonstrates consistently safe practices, a rate lower than in many other endemic regions [30]. Cultural beliefs, such as assuming a healthy-looking animal is safe, further blunt risk perception, underscoring the need for tailored, community-specific education.

In conclusion, Kazakhstan’s KAP profile reveals substantial room for improvement with a high potential return on investment. Effective interventions must pair demand-side education (targeting school-aged children and rural households) with supply-side enablers: vaccine subsidies, mobile veterinary clinics, and practical enforcement of Order No. 7-1/587. Such an integrated approach is essential to convert favorable attitudes into durable preventive practices, thereby reducing both incidence and mortality.

## 6. International Cooperation, Gaps and Opportunities for Improvement

The elimination of rabies in Central Asia is implemented within the framework of the tripartite agreement between the FAO, the World Organisation for Animal Health (WOAH), and the WHO, with the coordinating role of the WOAH Sub-Regional Representation in Astana. The key principle of this work is multisectoral coordination at all levels, carried out in accordance with the recommendations and strategies developed by international partners.

Significant contributions to building regional capacity are made by the European Union and the WHO Collaborating Centre for Rabies (Germany). Within the EU’s “Better Training for Safer Food” (BTSF) program, specialized trainings are funded, aimed at standardizing rabies surveillance and control methods. The Collaborating Centre provides scientific and technical support, acting as a WHO/WOAH Reference Laboratory and conducting training in modern diagnostic and monitoring techniques.

Coordination of efforts at the subregional level is carried out through platforms such as the Global Framework for the Transboundary Control of Animal Diseases (GF-TADs) [31]. This platform hosts regular meetings and seminars that bring together representatives of the veterinary services of Central Asian countries. An example is the Regional Seminar on the Integration of the One Health Concept into University Curricula (Almaty, October 2025), which addressed its application in specific technical activities.

To implement the approach at the national level, the WOAH Sub-Regional Representation organizes seminars for heads of territorial veterinary inspectorates, involving specialists from health authorities, emergency services, and border control agencies. During training, response algorithms for simulated epidemiological situations are practiced, leading to the development of practical recommendations for intersectoral collaboration.

Thus, the cooperation is built on a multi-level model: the EU provides the resource base, the Collaborating Centre provides expertise, and the WOAH Representation acts as the operator, ensuring coordination. This joint work fosters a unified epidemiological space and the development of common strategies, while the formulated recommendations are implemented at the level of regions, districts, and rural districts, which is key to the successful elimination of rabies in the region.

Kazakhstan’s rabies control system is built on a solid regulatory and operational foundation, but is constrained by critical gaps requiring urgent attention. The current surveillance system is predominantly passive and case-triggered, missing unreported events and wildlife reservoirs—particularly in remote rural districts—which leads to a significant underestimation of true prevalence. The annual oral rabies vaccination (ORV) program, distributing 736,000 baits [5], is undermined by insufficient bait density and suboptimal timing, limiting its population-level impact. Furthermore, efforts across sectors remain fragmented rather than integrated within a coordinated One Health framework, resulting in poor alignment between veterinary services, public health, municipalities, producers, and civil society. This is compounded by low community awareness; for instance, surveys indicate that 77% of rural residents do not fully value vaccination [11], a knowledge gap that amplifies spillover risk, especially from free-roaming dogs.

Addressing these gaps through targeted investments can significantly enhance control. According to the STOP-R strategy proposed at the international global rabies conference in 2015 [32], several key principles of anti-rabies strategy are already being implemented in Kazakhstan, providing a foundation to build upon. This includes progress on several fronts:

− Sociocultural (S) challenges persist. A major issue is the large population of stray dogs, with approximately 38,000 dog attacks on people recorded in 2024. A key discrepancy exists between national laws emphasizing animal birth control (ABC) and mass dog vaccination and the practices of many local authorities, who continue to contract “capture and destruction.”
− Technical (T): The country utilizes registered domestic and imported vaccines, and diagnostics are performed in accredited laboratories using ELISA and PCR. The necessary shift from passive to active surveillance and the introduction of mass vaccination and integrated bite case management (IBCM) are recognized priorities.
− Organizational and Political (OP): Interagency cooperation is regulated by the Veterinary Law, and there is coordination between veterinary and medical services. Politically, there is support for the global “Zero by 30” strategy, a national rabies elimination plan has been developed, and rabies is recognized as a priority disease.
− Resources (R): The state allocates significant funding (approx. 30 billion KZT annually), has increased the number of educational grants for veterinarians, and is equipping laboratories with modern equipment.

To fully leverage this foundation, targeted interventions are needed. Implementing GIS-enabled, near-real-time event mapping would improve the detection of outbreaks, including in high-risk border oblasts like Qostanay, which recorded six foci in 2025 [9]. The ORV program should be expanded and guided by habitat-suitability models (e.g., MaxEnt), while dog vaccination coverage must be raised to ≥70% [4] through subsidies that reduce out-of-pocket costs for owners and clinics. On the demand side, KAP-focused campaigns promoting PEP access and responsible dog ownership are essential. These measures should be supported by a standing, inter-agency One Health task force with a clear mandate, formal data-sharing protocols, and explicit performance targets, thereby strengthening the existing framework.

Collectively, these actions would reduce the estimated annual economic burden of USD 20.9 million [5] and prevent preventable deaths, making the WHO/WOAH “Zero by 30” goal an achievable objective for Kazakhstan.

## 7. Conclusions

Despite a century of control efforts, rabies remains an endemic threat in Kazakhstan, and transmission persists.

There is an urgent need to implement an integrated One Health approach to support disease prevention and control in the country, including improving targeted vaccination campaigns in high-risk areas and periods of time, enhancing and accelerating testing, and engaging with the public (including companion animal owners, farmers, and children) through preemptive education. Anchored in national Order No. 7-1/587 and the global “Zero by 30” strategy, this integrated package is not only feasible but also cost-effective, potentially preventing USD 20.9 million in annual losses. Only through the implementation of a policy based on sustained coordination, rather than just focusing on emergency response measures, will Kazakhstan be able to prevent and control dog-mediated human rabies deaths, with the ultimate goal of safeguarding public health and mitigating disease impact on the economy of the country.

## Figures and Tables

**Table 1:** Implementation of the rabies vaccination plan in Kazakhstan.

Category	Subcategory	Value	Unit	Note
Parenteral vaccination	Total number of doses	4.7 million	doses/year	2013–2015
	Total cost	1.7 million USD	USD/year	
	Cattle (cost)	1.4 million USD	USD/year	Price 0.35 USD/dose
	Distribution (South/East)	60%	%	
	Distribution (North)	20%	%	
	Distribution (West)	20%	%	
	Target coverage	70%	%	WOAH/WHO recommendation
Oral vaccination (ORV)	Number of baits	736 thousand	baits/year	Since the 2000s
	Total cost	719 thousand USD	USD/year	Price 0.97 USD/bait
	The recommended density	25–30	baits/km^2^	
Population control	Cost of trapping/euthanasia	3.4 million USD	USD/year	
Post-exposure prophylaxis (PEP)	Costs in Kazakhstan	9.1 million USD	USD/year	Globally, 29 million doses/year
Dog vaccination coverage	Qostanay region	21.1%	%	2024–2025
Reduction in morbidity	Livestock	68.1%	%	In the first half of 2025

## Data Availability

The data on rabies cases in Kazakhstan are available from the corresponding author upon a reasonable request.
